# Prognostic impact of extratumoral perineural invasion in patients with oral cavity squamous cell carcinoma

**DOI:** 10.1002/cam4.2392

**Published:** 2019-07-10

**Authors:** Li‐Yu Lee, Dante De Paz, Chien‐Yu Lin, Kang‐Hsing Fan, Hung‐Ming Wang, Chia‐Hsun Hsieh, Li‐Ang Lee, Tzu‐Chen Yen, Chun‐Ta Liao, Ching‐Hua Yeh, Chung‐Jan Kang

**Affiliations:** ^1^ Department of Pathology Linkou Chang Gung Memorial Hospital Taoyuan Taiwan, R.O.C; ^2^ Department of Head and Neck Oncology Group Linkou Chang Gung Memorial Hospital Taoyuan Taiwan, R.O.C; ^3^ Department of Plastic and Reconstructive Surgery Linkou Chang Gung Memorial Hospital Taoyuan Taiwan, R.O.C; ^4^ Department of Radiation Oncology Linkou Chang Gung Memorial Hospital Taoyuan Taiwan, R.O.C; ^5^ Department of Medical Oncology Linkou Chang Gung Memorial Hospital Taoyuan Taiwan, R.O.C; ^6^ Department of Otorhinolaryngology, Head and Neck Surgery Linkou Chang Gung Memorial Hospital Taoyuan Taiwan, R.O.C; ^7^ Department of Nuclear Medicine and Molecular Imaging Center Gung Memorial Hospital and Chang Gung University Taoyuan Taiwan, R.O.C; ^8^ Medicinal Botanicals and Health Applications Da Yeh University Changhua Taiwan, R.O.C; ^9^ Present address: Ph.D. Program of Biotechnology and Industry, College of Biotechnology and Bioresources Da-Yeh University 168 University Rd., Dacun Chang-Hua Taiwan Republic of China

**Keywords:** disease control, oral cavity squamous cell carcinoma, perineural invasion, prognosis, survival outcomes

## Abstract

**Purpose:**

Perineural invasion (PNI) is an adverse prognostic factor in patients with oral cavity squamous cell carcinoma (OCSCC). The American Joint Committee on Cancer Staging Manual, eighth edition, introduced a subdivision of PNI into two distinct forms, that is, extratumoral and intratumoral PNI (EPNI and IPNI, respectively). We designed the current study to assess whether EPNI and IPNI have different prognostic implications in terms of disease control and survival outcomes in patients with OCSCC.

**Materials and methods:**

We retrospectively examined 229 consecutive patients with OCSCC and PNI who underwent radical surgery between July 2003 and November 2016. EPNI and IPNI were identified in 76 and 153 patients, respectively. The 5‐year locoregional control (LRC), distant metastasis, disease‐free survival (DFS), and overall survival (OS) rates served as the main outcome measures.

**Results:**

Compared with patients showing IPNI, those with EPNI had a higher prevalence of worst pattern of invasion type‐5 (*P* < 0.001), alcohol consumption (*P* = 0.03), and close margins (*P* = 0.002). Univariate analysis revealed that EPNI was a significant predictor of 5‐year LRC (*P* = 0.024), DFS (*P* = 0.007), and OS (*P* = 0.034) rates. After allowance for potential confounders in multivariable analysis, ENPI was retained in the model as an independent predictor of 5‐year LRC (*P* = 0.028), DFS (*P* = 0.011), and OS (*P* = 0.034) rates.

**Conclusion:**

Compared with IPNI, the presence of EPNI in OCSCC portends less favorable outcomes. Patients with EPNI are potential candidates for definite aggressive treatment modalities aimed at improving prognosis.

## Graphical abstract

Extratumoral perineural invasion is significantly associated with poorer locoregional control, disease‐free survival and overall survival justifying its role as prognostic factor.

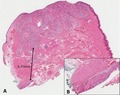

## 1 INTRODUCTION

Oral cavity squamous cell carcinoma (OCSCC) poses a significant health burden worldwide and represents one of the most common causes of cancer‐related mortality in Taiwan.^1^, ^2^ Despite the use of aggressive treatment modalities, advanced OCSCC remains associated with severe morbidity, high recurrence rates, and suboptimal survival outcomes. Recent advances in multidisciplinary team management hold promise for an integrated approach that takes into account all relevant treatment options—potentially resulting in tailored therapeutic strategies.^3^

In recent years, numerous clinicopathological risk factors—including perineural invasion (PNI)—have been studied in an effort to improve the prognostic stratification and treatment planning of patients with OCSCC.^4^ PNI—a form of cancer spread that can be broadly defined as evidence of tumor cell invasion within the nerve sheath and/or the epineurium—is mediated by neural cell adhesion molecule expression on the surface of tumor cells.^5^ Its prevalence has been reported to vary from 12% to 50% of all OCSCC specimens and its occurrence has been associated with higher rates of locoregional recurrence and poor survival outcomes.^6^, ^7^ Consequently, the National Comprehensive Cancer Network (NCCN) treatment guidelines for OCSCC maintain that PNI represents an indication for postoperative radiotherapy (RT) or concurrent chemoradiotherapy (CCRT).^8^ However, the prognostic impact of PNI has not been entirely elucidated owing to different methodological approaches used in its pathological assessment (resulting in a significant variability in terms of prevalence figures).^7^, ^9^, ^10^ Consequently, a more in‐depth analysis of PNI may help to gain further insights not only on its prognostic significance but also on its importance in the selection of locoregional or systemic treatments—potentially leading to better outcomes.^11^, ^12^

The American Joint Committee on Cancer (AJCC) Staging Manual, eighth edition, has recently proposed a subdivision of PNI into two distinct forms, that is, extratumoral and intratumoral PNI (EPNI and IPNI, respectively).^9^ We therefore designed the current retrospective study to assess whether EPNI and IPNI have different prognostic implications in terms of disease control and survival outcomes in a large homogeneous cohort of patients with OCSCC and PNI living in Taiwan, an endemic betel quid chewing area.

## 2 PATIENTS AND METHODS

### 2.1 Patients

We retrospectively reviewed the clinical records of consecutive, untreated patients with OCSCC (n = 1099) who underwent radical surgery in the Chang Gung Memorial Hospital (Taiwan) between July 2003 and November 2016. All pathological findings were thoroughly cross‐checked by two experienced head‐and‐neck pathologists using a dedicated checklist provided by our multidisciplinary tumor board. Upon identification of the tumor edge on each hematoxylin and eosin‐stained slice, the distance between the PNI focus and tumor edge was measured (in mm). Distances ≤ 0 mm and >0 mm were used to define intratumoral and extratumoral locations, respectively. Of 229 patients who had evidence of PNI, EPNI, and IPNI (according to the AJCC Staging Manual, eighth edition) were identified in 76 and 153 cases, respectively. All patients underwent an extensive presurgical evaluation that included (a) complete medical history and physical examination, (b) flexible fiberoptic pharyngoscopy, (c) complete blood count and routine laboratory testing, (d) computed tomography (CT) or magnetic resonance imaging (MRI) scans of the head and neck, (e) chest X‐ray, (f) bone scan, and (g) liver ultrasound. Patient staging was performed according to the AJCC Staging Manual, eighth edition.^13^ Follow‐up visits were continued until November 2018. All of the study patients were followed‐up for at least 24 months after surgery or until death. Ethical approval was granted by the local Institutional Review Board. The need for informed consent was waived owing to the retrospective nature of the study.

### 2.2 Surgery and adjuvant therapy

All primary tumors were excised with ≥1 cm safety margins (both peripheral and deep margins). Patients with cN‐ disease received neck dissection at the I–III levels. Neck dissection of I–V levels were performed in patients with cN + disease. Postoperative RT (60 Gy) was guided by the presence of pathological risk factors—which were classified using either the NCCN (before 2008) or the Chang Gung Memorial Hospital (as of 2008) guidelines.^8^ Specifically, RT was administered in the presence of pT4, pT1‐2N1, or pT3N1 disease (pN1 disease at neck level IV/V); 1‐2 mm close margins (in unresectable cases); and poor cell differentiation accompanied by a depth of invasion (DOI) ≥4 mm. We also offered RT to patients harboring two minor risk factors (ie, pN1, DOI ≥ 10 mm, 3‐4 mm close margins, poor cell differentiation, and perineural/lymphatic/vascular invasion). The radiation field comprised both the total tumor bed area (including 1‐ to 2‐cm margins) and the neck lymph nodes. CCRT (66 Gy) was offered to patients with extranodal extension (ENE), multiple metastases to neck lymph nodes, or positive margins. All of the patients with at least three of the above‐mentioned minor risk factors received CCRT as well.^14^, ^15^, ^16^

### 2.3 Statistical analysis

The 5‐year locoregional control (LRC), distant metastasis (DM), disease‐free survival (DFS), and overall survival (OS) rates served as the main outcome measures. Survival curves were constructed with the Kaplan‐Meier method (log‐rank test). Independent predictors of outcomes were identified with univariate and multivariable Cox proportional hazards regression models using a forward selection procedure.^8^ A total of 14 covariates (ie, worst pattern of invasion type‐5 [WPOI‐5], age, sex, preoperative alcohol drinking, betel quid chewing, cigarette smoking, pT, pN, ENE, tumor depth, tumor differentiation, margin status, lymphatic invasion, and vascular invasion) were entered into the multivariable model. Results are provided as hazard ratios with their 95% confidence intervals (CIs). Two‐tailed *P* < 0.05 were considered statistically significant.

## 3 RESULTS

### 3.1 Patients

A total of 229 patients were included in the study (212 men and 17 women; mean age: 52.37 years; range: 27‐89 years). The median follow‐up time in the entire study cohort was 45 months (mean: 48.76 months). Treatment modalities were as follows: surgery alone in 32 patients, surgery plus postoperative RT in 45 patients, and surgery plus postoperative CCRT in 152 patients.

Table 1 depicts the general characteristics of the study participants. Compared with patients showing IPNI, those with EPNI had a higher prevalence of worst pattern of invasion type‐5 ([WPOI‐5]; 56.6% vs 20.3%, respectively; *P* < 0.001), alcohol consumption in the preoperative period (81.7% vs 68.4%, respectively; *P* = 0.03), and close margins ≤ 4 mm (27.7% vs 11.1%, respectively; *P* = 0.002).

**Table 1:** General characteristics of the 229 study patients

Characteristics (n, %)	IPNI (n = 153) n (%)	EPNI (n = 76) n (%)	*P*
WPOI‐5			<0.001
No (155, 67.7)	122 (79.7)	33 (43.4)	
Yes (74, 32.3)	31 (20.3)	43 (56.6)	
Sex			0.437
Male (212, 92.6)	140 (91.5)	72 (94.7)	
Female (17, 7.4)	13 (8.5)	4 (5.3)	
Age, years			1.000
<65 (193, 84.3)	129 (84.3)	64 (84.2)	
>65 (36, 15.7)	24 (15.7)	12 (15.8)	
Alcohol consumption			0.030
No (52, 22.7)	28 (18.3)	24 (31.6)	
Yes (177, 77.3)	125 (81.7)	52 (68.4)	
Betel quid chewing			0.702
No (36, 15.7)	23 (15.0)	13 (17.1)	
Yes (193, 84.3)	130 (85.0)	63 (82.9)	
Cigarette smoking			0.836
No (30, 13.1)	21 (13.7)	9 (11.8)	
Yes (199, 86.9)	132 (86.3)	67 (88.2)	
Pathologic T status			0.174
pT1‐2 (72, 31.4)	53 (34.6)	19 (25.0)	
pT3‐4 (157, 68.6)	100 (65.4)	57 (75.0)	
Pathologic N status			1.000
pN0‐1 (134, 58.5)	90 (58.8)	44 (57.9)	
pN2‐3b (95, 41.5)	63 (41.2)	32 (42.1)	
ENE			1.000
No (157, 68.6)	105 (68.6)	52 (68.4)	
Yes (72, 31.4)	48 (31.4)	24 (31.6)	
Differentiation			0.599
Well/moderate (185, 80.8)	122 (79.7)	63 (82.9)	
Poor (44, 19.2)	31 (20.3)	13 (17.1)	
Tumor depth			0.534
<10 mm (64, 27.9)	45 (29.4)	19 (25.0)	
≥10 mm (165, 72.1)	108 (70.6)	57 (75.0)	
Margins status			0.002
≤4 mm (38, 16.6)	17 (11.1)	21 (27.7)	
>4 mm (191, 83.4)	136 (88.9)	55 (72.4)	
Lymphatic invasion			1.000
No (209, 91.3)	140 (91.5)	69 (90.8)	
Yes (20, 8.7)	13 (8.5)	7 (9.2)	
Vascular invasion			0.366
No (205, 89.5)	139 (90.8)	66 (86.8)	
Yes (24, 10.5)	14 (9.2)	10 (13.2)	
Treatment modality			0.419
S alone (21, 9.2)	15 (14.4)	6 (13.2)	
S plus RT/CCRT (208, 90.8)	138 (90.2)	70 (92.1)	

Abbreviations: CCRT, concurrent chemoradiotherapy; ENE, extranodal extension; EPNI, extratumoral perineural invasion; IPNI, intratumoral perineural invasion; RT, radiotherapy; S, surgery; WPOI‐5, worst pattern of invasion type‐5.

### 3.2 Five‐year outcomes

**Figure 1:**
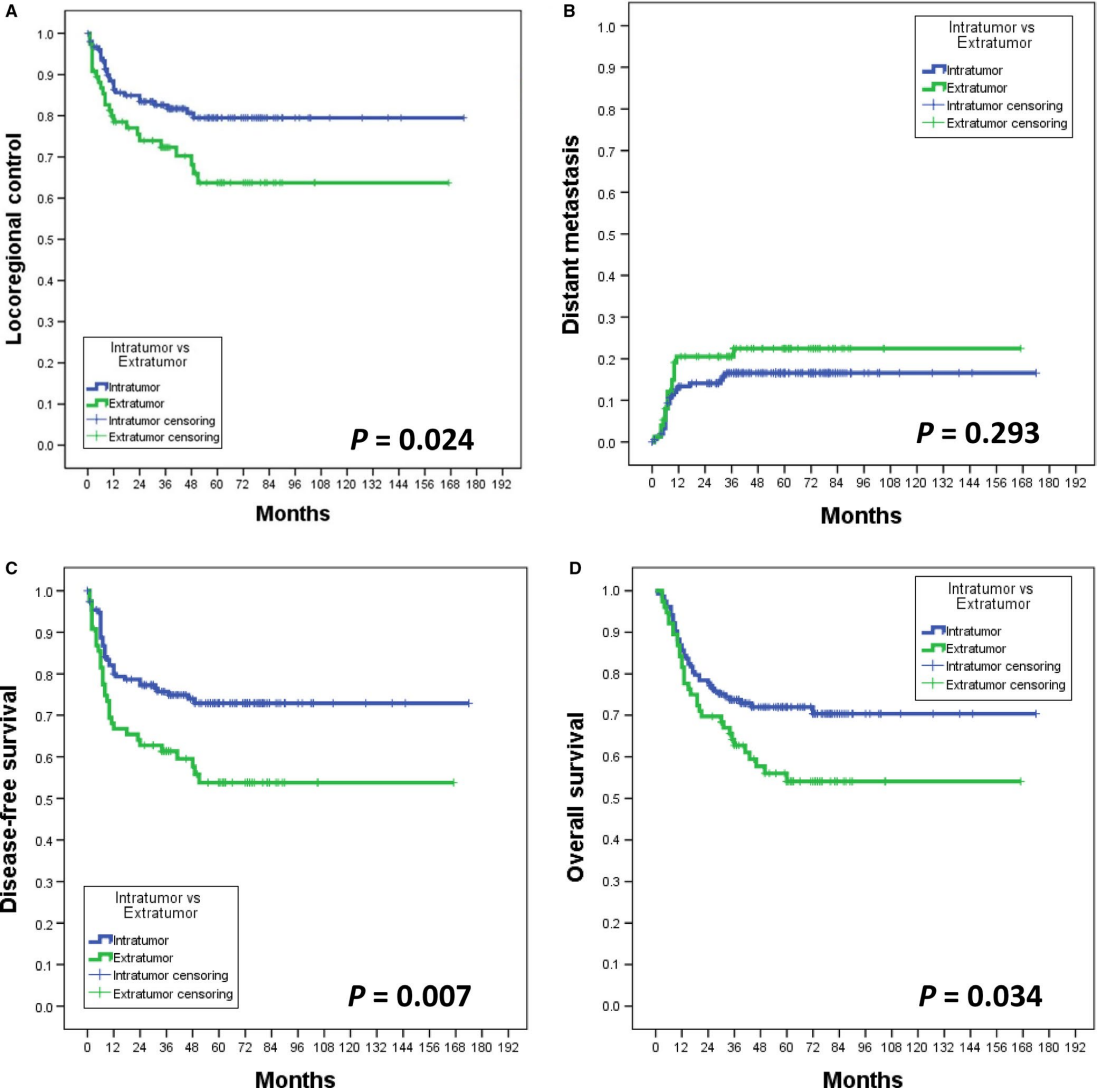
Survival and control curves

The following 5‐year rates were observed in the entire study cohort: LRC, 74.2%; DM, 16.2%; DFS, 75.6%; and OS, 65.8%. The 5‐year outcomes of patients with EPNI vs IPNI were as follows: LRC, 63.7% vs 79.5%, respectively, *P* = 0.024; DM, 22.5% vs 16.6%, respectively, *P* = 0.293; DFS, 53.8% vs 72.9%, respectively, *P* = 0.007; and OS, 54.1% vs 72.0%, respectively, *P* = 0.034 (Figure 1, panels A‐D).

### 3.3 Univariate and multivariable analyses of 5‐year outcomes

The results of univariate and multivariable analysis for 5‐year LRC, DM, DFS, and OS rates are presented in Tables 2 and 3, respectively. EPNI was a significant adverse predictor of 5‐year LRC (*P* = 0.024), DFS (*P* = 0.007), and OS rates (*P* = 0.034). The following variables were identified as significant predictors of 5‐year DM, DFS, and OS: pT3‐4 disease, pN2‐3b disease, ENE, poor differentiation, lymphatic invasion, and vascular invasion. Moreover, close margins≤4 mm were a significant predictor for the 5‐year DM rate, whereas a tumor depth ≥10 mm was associated with both 5‐year DM and OS rates (Table 2). The results of multivariable analysis revealed that EPNI was an independent adverse prognostic factor for 5‐year LRC, DFS, and OS rates. In addition, the following independent risk factors were identified: pT3‐4 disease, ENE, poor differentiation, and lymphatic invasion for the 5‐year DM rate; ENE, poor differentiation, and lymphatic invasion for the 5‐year DFS rate; pT3‐4 disease, pN2‐3b disease, poor differentiation, and lymphatic invasion for the 5‐year OS rate (Table 3).

**Table 2:** Univariate analysis of 5‐year control and survival rates in the 229 study patients

Risk factor (n)	Locoregional control % (n event)	*p*	Distant metastasis % (n event)	*p*	Disease‐free survival % (n event)	*p*	Overall survival % (n event)	*p*	
Perineural invasion		0.024		0.293		0.007		0.034	
IPNI (153)	81.7 (28)		15.7 (24)		74.5 (39)		71.9 (43)		
EPNI (76)	68.4 (24)		21.1 (16)		56.6 (33)		56.6 (33)		
WPOI‐5		0.124		0.385		0.035		0.257	
No (155)	80.0 (31)		16.1 (25)		72.9 (42)		69.0 (48)		
Yes (74)	71.6 (21)		10.3 (15)		59.5 (30)		62.2 (28)		
Sex		0.471		0.462		0.347		0.436	
Male (212)	77.8 (47)		17.0 (36)		69.3 (65)		67.5 (69)		
Female (17)	70.6 (5)		23.5 (4)		58.8 (7)		58.8 (7)		
Age, years		0.297		0.614		0.591		0.144	
<65 (193)	78.2 (42)		17.1 (30)		68.9 (60)		68.4 (61)		
≥65 (36)	72.2 (10)		19.4 (7)		67.7 (12)		58.3 (15)		
Alcohol consumption		0.861		0.559				0.421	
No (52)	78.8 (11)		19.2 (10)		67.3 (17)	0.719	63.5 (19)		
Yes (177)	76.8 (41)		16.9 (30)		68.9 (55)		67.8 (57)		
Betel quid chewing		0.910		0.610		0.641		0.980	
No (36)	77.8 (8)		13.9 (5)		72.2 (10)		66.7 (12)		
Yes (193)	77.2 (44)		18.1 (35)		67.9 (62)		66.8 (64)		
Cigarette smoking		0.982		0.681		0.861		0.748	
No (30)	76.7 (7)		20.0 (6)		70.0 (9)		70.0 (9)		
Yes (199)	77.4 (45)		17.1 (34)		68.3 (63)		66.3 (67)		
Pathologic T status		0.320		0.001		0.016		0.001	
pT1‐2 (72)	79.2 (15)		5.6 (4)		77.8 (16)		80.6 (14)		
pT3‐4 (157)	76.4 (37)		28.9 (36)		64.3 (56)		60.5 (62)		
Pathologic N status		0.267		<0.001		<0.001		<0.001	
pN0‐1 (134)	78.4 (29)		6.7 (9)		76.1 (32)		79.1 (28)		
pN2‐3b (95)	75.8 (23)		32.6 (31)		57.9 (40)		49.5 (48)		
ENE		0.390		<0.001		<0.001		<0.001	
No (157)	77.7 (35)		8.3 (13)		75.2 (39)		75.2 (39)		
Yes (72)	76.4 (17)		37.5 (27)		54.2 (33)		48.6 (37)		
Differentiation		0.755		<0.001		0.020		0.002	
Well/moderate (185)	76.2 (44)		13.0 (24)		71.4 (53)		70.8 (52)		
Poor (44)	81.8 (8)		36.4 (16)		56.8 (19)		50.0 (22)		
Tumor depth		0.770		0.004		0.262		0.025	
<10 mm (64)	73.4 (17)		6.2 (4)		71.9 (18)		76.6 (15)		
≥10 mm (165)	78.8 (35)		21.8 (36)		67.3 (54)		63.0 (61)		
Margins status		0.294		0.046		0.298		0.265	
≤4 mm (38)	71.1 (11)		28.9 (11)		60.5 (15)		60.5 (15)		
>4 mm (191)	78.5 (41)		15.2 (29)		70.2 (57)		68.1 (61)		
Lymphatic invasion		<0.001		<0.001		<0.001		<0.001	
No (209)	79.9 (42)		13.9 (29)		71.8 (59)		70.3 (77)		
Yes (20)	50.0 (10)		55.0 (11)		35.0 (13)		30.0 (13)		
Vascular invasion		0.138		0.022		0.031		0.044	
No (205)	78.5 (44)		15.6 (32)		70.7 (60)		68.8 (64)		
Yes (24)	66.7 (8)		33.3 (8)		50.0 (12)		50.0 (12)		

Abbreviations: ENE, extranodal extension; EPNI, extratumoral perineural invasion; IPNI, intratumoral perineural invasion; WPOI‐5, worst pattern of invasion type‐5.

**Table 3:** Multivariable analysis of 5‐year control and survival rates in the 229 study patients

Risk factor (n)	Locoregional control *p*; HR (95% CI)	Distant metastasis *p*; HR (95% CI)	Disease‐free survival *p*; HR (95% CI)	Overall survival *p*; HR (95% CI)
EPNI (n = 76)	0.028 1.8 (1.1 to 3.2)	ns	0.011 1.8 (1.1 to 2.9)	0.034 1.6 (1.0 to 2.6)
Pathologic T3‐4 (n = 157)	ns	0.033 3.2 (1.1 to 9.4)	ns	0.039 1.9 (1.0 to 3.5)
Pathologic N2‐3b (n = 95)	ns	ns	ns	0.003 2.2 (1.3 to 3.6)
ENE (n = 72)	ns	<0.001 3.6 (1.8 to 7.3)	0.007 1.9 (1.2 to 3.1)	ns
Poor differentiation (n = 44)	ns	<0.001 3.8 (2.0 to 7.3)	0.013 2.0 (1.2 to 3.4)	0.001 2.3 (1.4 to 3.9)
Lymphatic invasion (n = 20)	<0.001 3.9 (2.0 to 7.9)	<0.001 4.3 (2.1 to 9.0)	<0.001 3.5 (1.9 to 6.6)	0.003 2.6 (1.4 to 4.8)

Abbreviations: CI, confidence interval; ENE, extranodal extension; EPNI, extratumoral perineural invasion; HR, hazard ratio; ns, not significant.

## 4 DISCUSSION

PNI is generally considered as an adverse prognostic factor in patients with OCSCC and its presence is deemed to pose an indication for adjuvant treatment.^17^, ^18^ Although the clinical benefits of RT in patients with PNI have been repeatedly demonstrated,^10^, ^19^ the exact prognostic significance of PNI in OCSCC remains incompletely understood—potentially resulting in treatment discrepancies. In this regard, the current NCCN guidelines recommend the use of adjuvant RT/CCRT in intermediate‐risk OCSCC—a category which comprises tumors with substantial PNI (ie, PNI of large nerves or evidence of tumor infiltrates not limited to a low number of small sensory branches).^8^ However, the Chang Gung Memorial Hospital guidelines maintain that the presence of PNI alone does not justify the use of adjuvant therapy—with radical surgery being considered sufficient.^20^

Previous studies have shown that PNI can be detected in both primary and recurrent tumors, irrespective of their histological grading. In addition, tumor thickness and nodal status have been associated with the presence of PNI.^21^ These observations notwithstanding, discrepant data exist on the prognostic significance of PNI in terms of locoregional recurrence and survival.^7^, ^10^ Such inconsistencies may at least in part be explained by methodological limitations inherent to the published studies (eg, small sample size and/or inclusion of patients with various disease stages and tumors arising from different subsites).^22^, ^23^ According to the classification proposed by Miller et al,^9^ the extent of PNI should be assessed by measuring the distance (in millimeters) from each focus of invasion to the tumor edge (which is set at 0 mm). The authors observed a trend for a longer DFS for patients with strictly intratumoral PNI compared with those showing additional peripheral and extratumoral PNI.^9^ In light of these observations, the AJCC Staging Manual, eighth edition, has proposed a subdivision of PNI into two distinct categories (IPNI vs EPNI).^13^

To the best of our knowledge, this is the largest study to date to specifically examine the prognostic impact of IPNI vs EPNI in patients with OCSCC. Our results revealed that the presence of EPNI was associated with a higher likelihood of WPOI‐5 and surgical margins ≤4 mm compared with IPNI. These observations can be explained by the higher invasive potential of tumors showing EPNI—even when excised with ≥1 cm safety margins.^24^ WPOI‐5—defined by small tumor islands or satellite tumors located at least 1 mm away from the main neoplasm—portends a high risk of local recurrences and poor OS.^25^, ^26^ Notably, this pattern reflects a pronounced tumor invasiveness related to an altered expression of extracellular matrix remodeling genes.^27^

In light of these observations, EPNI can be considered as a proxy of locally aggressive tumor behavior—which may in turn explain its unfavorable impact on 5‐year LRC, DFS, and OS rates observed in our study. Although patients with ENPI did not differ from those with IPNI in terms of 5‐year DM, the former group was characterized by a higher risk of locoregional recurrences and a lower DFS compared with the latter. In our study, EPNI was identified as an independent adverse risk factor for LRC—which can also account for the similar unfavorable impact observed on DFS and OS. As far as distant metastases are concerned, we have previously shown that their main predictors included pT3‐4 disease, ENE, lymphatic invasion, and poor differentiation.^28^ Taken together, these data suggest that neither PNI in general nor its subclassification (ENPI vs INPI) is a key determinant of distant metastatic spread (being rather a marker of local tumor invasiveness).

Although EPNI is associated with poorer LRC compared with IPNI, we believe that the indications for adjuvant RT/CCRT in presence of PNI should be tailored at the individual level by taking into account the presence of comorbidities, age, and the patient's preferences.^29^ Previous studies have shown that cisplatin given concurrently with postoperative RT improves LRC and DFS rates in patients with high‐risk squamous cell carcinoma of the head and neck.^17^, ^18^ Although the major benefits of CCRT have been observed in presence of positive margins and/or ENE, a trend toward better outcomes was also evident for patients harboring minor risk factors (including PNI).^30^ In line with this possibility, our data indicate that patients with EPNI do not only require a thorough resection of the primary tumor but also a comprehensive management of the neck lymph nodes alongside with adjuvant RT (to improve LRC and survival rates). In our study, six patients with evidence of EPNI were treated with surgery only (Table 1). The local recurrence rate for this subgroup was 50% (ie, 3 events in 6 patients) and higher than that observed in the IPNI group (20%, ie, 3 events in 15 patients). Although such difference was not statistically significant owing to the small sample size, this observation corroborates the notion that EPNI portends a high local recurrence rate in patients with OCSCC. Although PNI is considered a minor risk factor for postoperative adjuvant treatment according to our guidelines, the present study demonstrates that EPNI is an independent adverse prognostic factor of 5‐year LRC, DFS, and OS rates. We therefore believe that EPNI should be considered as a major risk factor and an indication for RT in intermediate‐risk patients who do not harbor other adverse prognosticators.

From a pathophysiological standpoint, PNI appears to be mediated by the expression of neural cell adhesion molecules on the surface of OCSCC tumor cells (which facilitate their spread through the perineural tissue).^7^ A better understanding of the molecular underpinnings of PNI in OCSCC may facilitate the development of more effective therapeutic strategies in the next future.^21^, ^31^

## 5 LIMITATIONS

Our study is limited by its retrospective design and the sole inclusion of patients living in a betel quid chewing endemic area. The question as to whether our data may be generalizable to other countries remains open. International multicenter studies conducted in non‐betel quid chewing areas are required to confirm and expand our findings.

## 6 CONCLUSIONS

Compared with IPNI, the presence of EPNI in patients with OCSCC portends a less favorable prognosis and is an independent adverse predictor of 5‐year LRC, DFS, and OS rates. Patients with EPNI are potential candidates for definite aggressive treatment modalities (including adjuvant radiotherapy) aimed at improving clinical outcomes.

## CONFLICTS OF INTEREST

The authors have no conflicts of interest to disclose.
