# Supplementary material for: Prognostic impact of extratumoral perineural invasion in patients with oral cavity squamous cell carcinoma
Source: Cancer Med. 2019 Jul 10;8(14):6185–94. doi: 10.1002/cam4.2392 (PMC6797567; doi:10.1002/cam4.2392)
Supplement: Supplementary file 1 [file CAM4-8-6185-s001.pdf]

Supplementary Figures

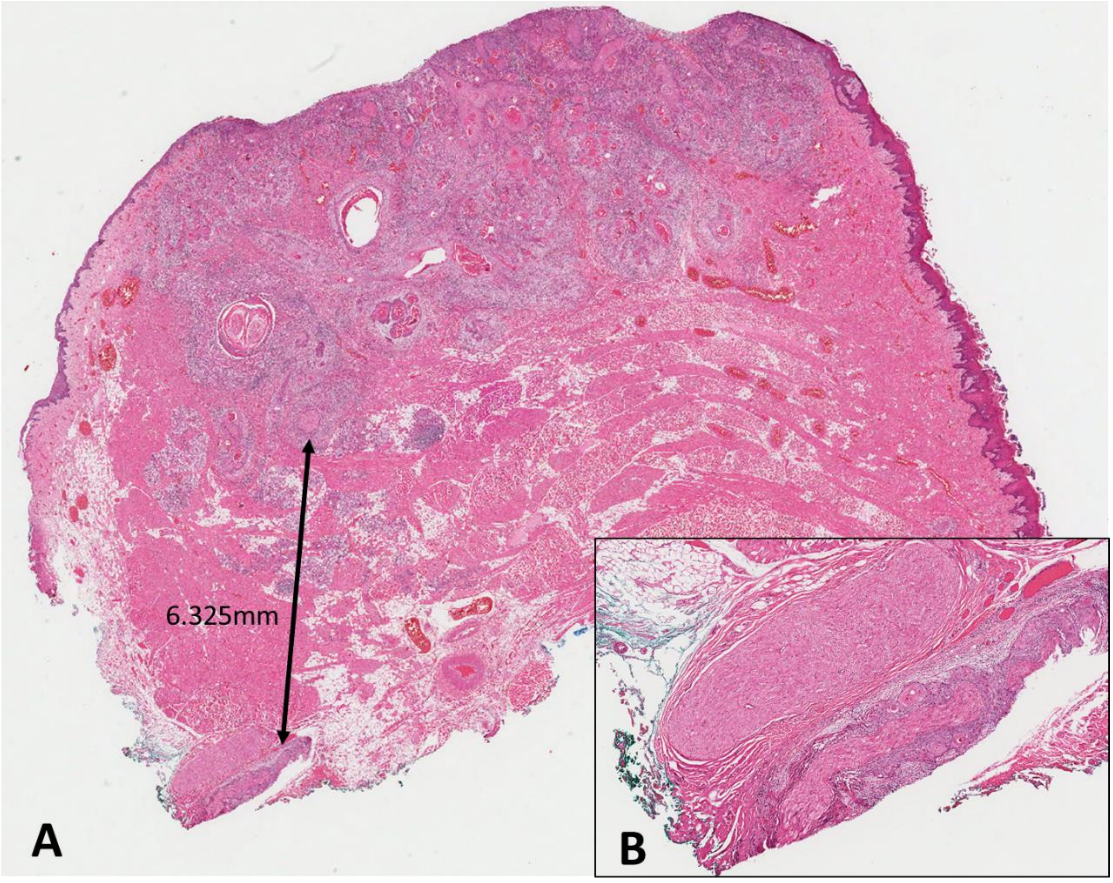

Figure 1. Panel A. Scan view of extratumoral perineural invasion (EPNI). The distance between EPNI and tumor edge was 6.325 mm. Panel B. EPNI was close to the inked resection margin (200 × magnification).

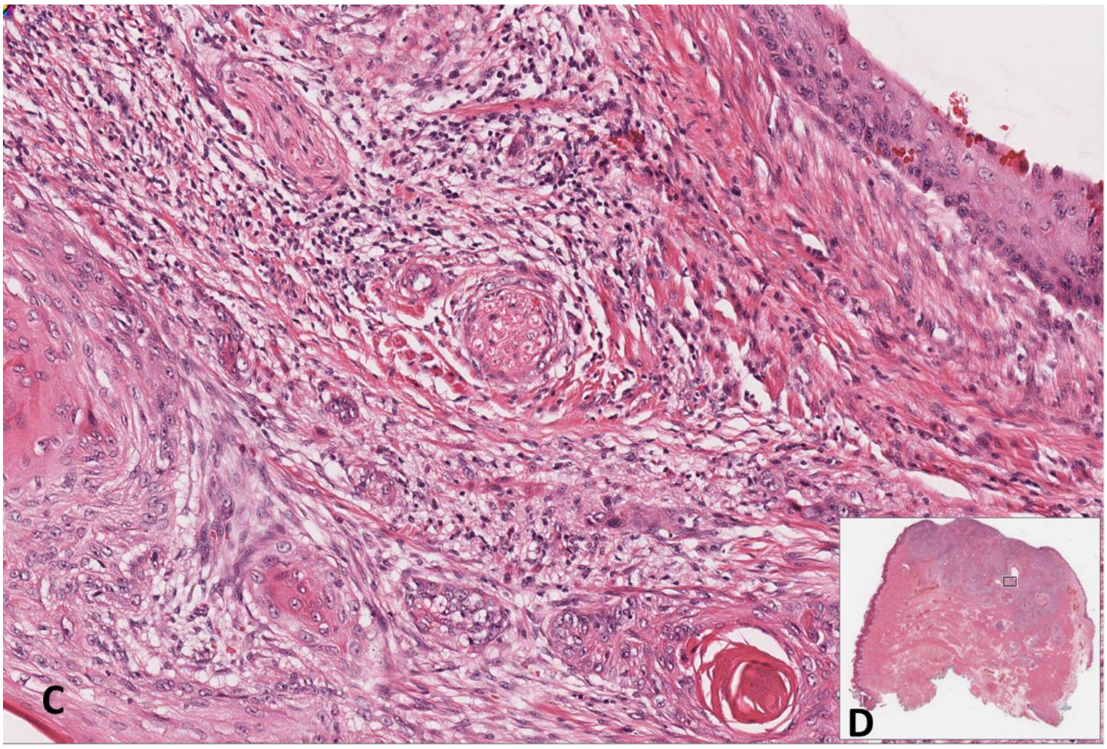

Figure 2. Panel C. Image of intratumoral perineural invasion (IPNI) obtained from the small black rectangular frame within the scan view (200 × magnification). Panel D. Scan view of IPNI.

Figure legends

Figure 1. Panel A. Scan view of extratumoral perineural invasion (EPNI). The distance between EPNI and tumor edge was 6.325 mm. Panel B. EPNI was close to the inked resection margin (200 × magnification).

Figure 2. Panel C. Image of intratumoral perineural invasion (IPNI) obtained from the small black rectangular frame within the scan view (200 × magnification). Panel D. Scan view of IPNI.
